# Real World Outcomes of a Condensed Classroom‐Based Cognitive Behavioral Therapy for Cancer‐Related Cognitive Impairment

**DOI:** 10.1002/pon.70409

**Published:** 2026-03-14

**Authors:** Shelly Kucherer, Hailey B. King, Robert J. Ferguson

**Affiliations:** ^1^ Department of Psychiatry University of Pittsburgh School of Medicine Pittsburgh Pennsylvania USA; ^2^ Biobehavioral Cancer Control Program UPMC Hillman Cancer Center Pittsburgh Pennsylvania USA; ^3^ Tulane University School of Medicine New Orleans Louisiana USA; ^4^ Department of Medicine, Division of Hematology/Oncology University of Pittsburgh School of Medicine Pittsburgh Pennsylvania USA; ^5^ Department of Psychology and Biobehavioral Sciences Cancer Control and Survivorship Program St Jude Children's Research Hospital Memphis Tennessee USA

**Keywords:** cancer supportive care, cancer‐related cognitive impairment, cognitive‐behavioral therapy, stepped‐care

## Abstract

**Background:**

Cancer‐related cognitive impairment (CRCI) affects a large proportion of cancer survivors and adversely affects quality of life (QoL). While there is no established CRCI treatment, a cognitive‐behavioral therapy (CBT), Memory and Attention Adaptation Training (MAAT), has evidence of efficacy in improving self‐reported and objective neurocognitive outcomes. However, not all people with CRCI require a full course of MAAT (8 visits).

**Aims:**

We condensed MAAT into an educational class (4 1‐h meetings) as a “stepped care” approach to address CRCI in a busy clinical oncology care setting. Our goal with this quality improvement project was to offer brief, accessible CRCI care and evaluate self‐reported cognitive function.

**Methods:**

“MAAT‐Class” utilizes discussion, problem‐solving and a PowerPoint presentation emphasizing CRCI education, self‐awareness, self‐regulation/emotional coping, and compensatory strategies. All participants use a MAAT workbook. Self‐report measures assessing cognitive symptoms, emotional function, coping and sleep were administered before and after MAAT‐Class.

**Results:**

Twenty‐two adult participants with diverse cancer diagnoses (breast, leukemia/lymphoma, CNS tumor) provided complete data. Significant improvements were observed in Perceived Cognitive Impairments (*p* < 0.05; *d* = 0.79), Impact on QoL (*p* < 0.05; *d* = 0.39), Perceived Cognitive Abilities (*p* < 0.05; *d* = 0.88), relaxation skills (*p* < 0.001; *d* = 0.77) and coping confidence (*p* < 0.001; *d* = 0.77).

**Conclusions:**

A condensed classroom‐based version of MAAT demonstrated improvements in perceived cognitive impairments, QoL and coping. This first‐step clinical educational approach could potentially and efficiently help inform patients with CRCI and improve QoL outcomes. Administering MAAT‐Class via telehealth could potentially help survivorship care access. However, evaluation with a randomized study is necessary to definitively answer questions of efficacy.

## 1 Background

Cancer‐related cognitive impairment (CRCI) is a set of persistent mild to moderate decrements in memory and attention that affect approximately half of all cancer survivors [1]. CRCI can have adverse effects on work, social function and educational attainment that leads to long‐term, detrimental economic and health outcomes [1]. Owing to continued success of screening, early detection and improved cancer therapies, annual cancer mortality rates continue to drop, resulting in over 18 million Americans today who have had a cancer diagnosis in their lifetime [2]. In view of the large number of individuals potentially affected by CRCI, addressing the problem is a survivorship care priority.

Over the last 2 decades, non‐pharmacologic treatments for CRCI have been developed and evaluated but none are established as standard care [1, 3, 4]. One treatment with demonstrated efficacy among breast cancer survivors is Memory and Attention Adaptation Training (MAAT), a manualized cognitive behavioral therapy (CBT) [5, 6, 7]. MAAT consists of eight weekly 45‐min visits covering CRCI education, self‐awareness of “at risk” situations where cognitive problems in daily life may occur, modification of causal attributions that may be biased toward cancer‐related causes of memory difficulty versus more controllable factors such as inattention/organization, and training in compensatory strategies [8]. Survivors work with a clinician and a workbook [9] over the course of treatment. MAAT has demonstrated efficacy with telehealth well before the COVID‐19 pandemic, which reduces survivor travel and cost burdens [5, 10].

However, not all cancer survivors require a full course of CBT. Many cancer centers, especially community centers or affiliates, may not have staff to meet survivorship care demands which may cause waitlists [11]. To address this problem, we condensed MAAT into a 4‐session “classroom” format and evaluated initial effectiveness at improving survivor cognitive outcomes. The intent of the class is to provide an overview and introduction to CRCI treatment in a stepped care fashion to ease cancer survivorship care waitlists [12] and help survivors determine if more treatment is necessary. Our aim with this clinical quality improvement project was to evaluate MAAT‐Class in a real‐world oncology care setting and determine if it could substantively improve self‐reported cognitive function. We present observed outcomes of the brief MAAT‐Class format as part of CRCI survivorship care.

## 2 Methods

### 2.1 Participants

Participants were oncology patients who responded to cancer center outpatient oncology service waiting area flyers and/or discussion with staff oncologists, psychiatrists, and psychologists. Flyers outlined the class format and advised that the class was intended for mild to moderate problems with daily memory. Those with more severe impairments (e.g., those people who require close supervision) or psychiatric illness (e.g., severe depression or psychotic disorder) were advised to seek specialty care. Patients at various phases of their cancer experience, including those currently undergoing primary treatment and those who completed treatment were invited. Patients registered with our clinical service, were welcome to invite family members, and were not billed for this educational offering. Participating patients and family members were not required to show for all individual sessions and were allowed to repeat another MAAT‐Class offered in the future.

### 2.2 MAAT‐Class

MAAT‐Class was condensed in content from the 8‐visit MAAT CBT format into four 1‐h sessions with an accompanying PowerPoint presentation. All classes were conducted by a physician Psycho‐Oncology fellow (SK) who was trained and supervised by the senior author (RJF). The PowerPoint‐based discussion covered topics of CRCI education and re‐attribution (i.e., raising awareness that memory failures may be due to modifiable causes such as inattention or stress responding vs. less modifiable causes such as cancer diagnosis), self‐awareness or self‐monitoring of “at‐risk” situations where memory problems are more prone to occur, stress management (cognitive modification, sleep quality improvement, applied self‐regulation/relaxation) and cognitive compensatory strategies. Participants were encouraged to foster a supportive environment and engage in discussions that included reporting examples of applying strategies in daily life, brainstorming with other participants, and problem‐solving for implementation. Each was provided a MAAT Survivor Workbook to select and apply the most relevant topics to their personal circumstances between sessions. All participants pre‐registered and signed attendance sheets.

### 2.3 Measures

Prior to the first class and following the last, participants were requested to complete validated measures on cognitive symptoms and impact they had on quality of life as assessed by the Functional Assessment of Cancer Therapy‐Cognitive scale, Version 3 (FACT‐Cog) [13]. The FACT‐Cog v.3 consists of 4 separate scales that are scored individually, and include Perceived Cognitive Impairments (PCI; the principal outcome of the present project); Impact on Quality of Life (IQoL), Perceived Cognitive Abilities (PCA), and Comments From Others (CFO; a measure of perceived statements from others, such as close family members, about cognitive function). Higher scores on all FACT‐Cog scales indicate better function. Other measures of quality of life (QoL) include, anxiety (Generalized Anxiety Disorder‐7; GAD‐7) [14], depression (Patient Health Questionnaire‐9; PHQ‐9) [15], perceived stress (Perceived Stress Scale; PSS) [16], social support (MOS Social Support Survey‐4‐item) [17], along with relaxation skills, awareness of relaxation, and coping confidence as assessed by the Management of Current Stress (MOCS) scale [18]. Likert ratings (0 = absent‐10;  = worst possible over the previous week) were used to assess sleep disturbance. The core of this battery of measures (except for the FACT‐Cog) was used in other workshops at the cancer center.

### 2.4 Statistical Analysis

Analyses included descriptive summaries of demographic and outcome variables for distribution and central tendency. Correlational analyses were conducted with age and outcome measures at baseline to determine potential covariates. Repeated measures analysis of covariance (ANCOVA), controlling for age, was conducted for PCI with correlated *t*‐tests computed to determine group change from baseline to post‐MAAT‐Class. All tests were set a *α* < 0.05 and Cohen's *d* size of effect was reported on outcomes [19] The University of Pittsburgh Medical Center (UPMC) Clinical Improvement Review Committee Approved this project (project ID 396). All analyses were conducted with SPSS Version 29.0.2.0.

## 3 Results

**FIGURE 1:**
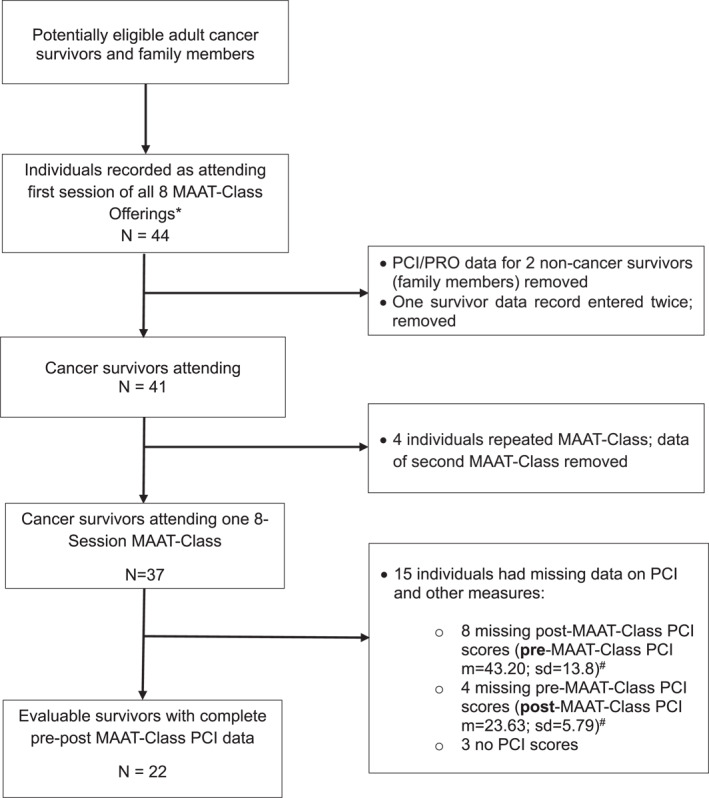
Flow diagram of PCI and patient‐reported outcome data collection. Data collected from 8 MAAT‐Classes. Each MAAT‐Class consisted of 4 weekly sessions. m, mean; PCI, FACT‐Cog Perceived Cognitive Impairment scale; sd, standard deviation.

Across 8 separate offerings of the 4‐session MAAT‐Class, a total of 41 individuals attended the first of 4 sessions while 32 attended the fourth, with an overall non‐attendance of 22%. An average of 4 participants attended individual MAAT‐Class sessions (range 2–7/session). Twenty‐two cancer survivors completed both pre‐ and post‐ PCI and thus had evaluable outcome data that was used in analyses. Individuals with incomplete responses to outcome measures or who did not turn in baseline or post‐MAAT‐Class measures were excluded. Two (non‐cancer) family members who completed measures were excluded and one survivor had repeated data entered in the data set which was removed. Four participants attended 2 MAAT‐Classes, and only pre‐post data on their first MAAT‐Class attended was included in analyses. The flow diagram of data acquisition is seen in Figure 1.

The mean age of survivors with complete data (*N* = 22) was 63.08 years and were on average 4.17 years post‐diagnosis (Table 1). Nine (41%) were breast cancer survivors, 6 were lymphoma/leukemia survivors (27%) followed by 2 individuals with ovarian cancer, 1 with primary brain tumor and individuals with head and neck, peritoneal, gallbladder, thyroid and prostate cancer, respectively. Nineteen were women (86%) and 17 (77%) were married or living with a partner. Eleven individuals (50%) reported either trade, college or some post‐secondary education while 9 (41%) reported post‐college graduate education with 2 reporting less than a high school degree.

**TABLE 1:** Participant characteristics.

Variable	MAAT‐class participants (*N* = 22)	Participants with missing data (*n* = 15)
Age in years (*M*/SD)	63.08 (12.49) range = 40–83	56.97 (12.74) range = 38–84
Sex		
Male	3	4
Female	19	11
Level of education		
High school or less	2	2
Some college or college degree, trade school	11	11
Post‐graduate	9	2
Employment		
Working	5	3
Not working or retired	17	12
Marital status		
Married or living with a partner	17	11
Not married	5	4
Cancer type		
Breast	9	5
Gastrointestinal		3
Lymphoma/leukemia	6	4
Primary brain tumor	1	1
Ovarian	2	1
Head and neck	1	
Prostate	1	
Gallbladder	1	
Primary peritoneal	1	
Sarcoma		1
Thyroid^a^	1	
Years since diagnosis (years) *M* (SD)	4.17 (4.28) range = < 1 to 17	3.2 (1.75) range = < 1 to 9.9

^a^ One survivor reported both breast and thyroid cancer accounting for the MAAT‐Class (*N* = 22) column total of 23.

Fifteen (68.20%) participants reported cognitive symptoms with a significant negative impact on QoL prior to MAAT‐Class (FACT‐Cog IQoL score of ≤ 10) [20] and on average reported mild‐moderate anxiety (GAD = 7.22) [14] and depressive symptoms (PHQ‐9 score of 8.47) [15]. In terms of overall level of cognitive impairment (FACT‐Cog PCI), a mean score of 33.80 is consistent with other MAAT research at pre‐treatment and survey research using the FACT‐Cog as a primary measure [5, 20].

Age was found to correlate significantly with PCI at baseline (*r* = 0.52; *p* < 0.05). Age did not correlate with other outcomes. Adjusting for age, statistically significant improvements post‐MAAT‐Class were observed in PCI *F*(1,22) = 5.53 *p* = 0.029 with a large effect size of −0.794 (negative integer does not alter magnitude). Significant improvements were observed for IQoL score, *t*(21) = −1.81, *p* = 0.042; *d =* −0.387; PCA, *t*(21) = −2.10, *p* = 0.024; relaxation skills, *t*(20) = −3.50, *p* = 0.001; *d* = −0.766, awareness of relaxation *t*(20) = −2.90, *p* = 0.004; *d* = −0.634, and coping confidence *t*(21) = −3.60, *p* = 0.001; *d* = −0.768 with medium to large effects (Table 2).

**TABLE 2:** Outcomes. Complete PCI data *N* = 22 linear interpolation *N* = 37.

	Baseline *m* (sd)	Post‐MAAT *m* (sd)	*p*	*d*	Baseline *m* (sd)	Post‐MAAT *m* (sd)	*p*
^a^ Fact‐Cog PCI 0–72	33.80 (14.31)	39.64 (12.06)	**0.029**	−0.794	36.20 (13.07)	37.29 (10.57)	0.27
Fact‐Cog IQoL 0–16	7.24 (5.08)	8.50 (4.01)	**0.042**	−0.387	8.21 (4.55)	7.89 (3.95)	0.332
Fact Cog CFO 0–16	12.90 (3.43)	12.33 (3.55)	0.232	−0.27	13.27 (2.78)	12.29 (4.10)	0.069
Fact‐Cog PCA 0–28	12.14 (5.22)	13.92 (5.00)	**0.024**	−0.882	11.82 (5.12)	13.05 (5.22)	0.071
GAD‐7 (anxiety) 0–21	7.22 (5.55)	6.47 (4.95)	0.136	−0.187	7.81(6.14)	7.21 (4.40)	0.219
PHQ‐9 (depression) 0–32	8.47 (4.91)	7.82 (5.53)	0.076	−0.115	8.85 (5.94)	7.79 (5.52)	0.075
PSS 0–16	6.55 (2.94)	6.18 (2.44)	0.203	−0.243	6.70 (3.04)	6.9 (2.61)	0.496
Social support 0–16	11.45(4.33)	11.73 (4.26)	0.327	−0.515	10.66 (4.59)	11.10 (3.10)	0.267
Relaxation skills 2–10	4.91 (1.87)	5.86 (1.31)	**0.001**	−0.766	4.73 (1.73)	5.46 (1.51)	**0.004**
Awareness of relaxation 3–15	8.24 (1.87)	9.62 (2.22)	**0.004**	−0.634	8.54 (2.17)	9.42 (2.03)	**0.021**
Coping confidence 5–25	13.27 (3.71)	15.05 (4.06)	**0.001**	−0.768	13.50 (3.10)	15.07 (3.70)	**0.001**
Sleep disturbance 0–10	3.76 (3.03)	3.29 (2.87)	0.176	0.208	3.10 (3.28)	4.30 (2.91)	0.277

*Note:* Cohen's *d* effect size is for paired‐sample comparisons and can be inflated with the standard deviation of the differences in the denominator— interpretations adjusted to this context are: Small, 0.20–0.39; Medium, 0.40–0.69; Large, 0.70–0.99; Very large, > 1.00. Relaxation Skills, Awareness of Relaxation, and Coping Confidence are scales from the Management of Current Stress (MOCS) scale. Higher scores denote better function. All FACT‐Cog scales are interpreted as higher scores = better function. Higher scores, including the sleep disturbance 0–10 Likert scale, denote higher distress. Bold = statistically signficant pre‐post differences (< 0.05).

Abbreviations: CFO, Comments From Others; FACT‐Cog, Functional Assessment of Cancer Therapy, Cognitive v.3—The 4 FACT‐Cog Scales are PCI=Perceived Cognitive Impairments (18 item); GAD‐7, Generalized Anxiety Disorder, 7‐Item; IQoL, Impact on Quality of Life.PCA, Perceived Cognitive Abilities; PHQ‐9, Patient Health Questionnaire, 9‐Item; PSS, Perceived Stress Scale.

^a^ ANCOVA with age as covariate.

### 3.1 Intent‐to‐Treat (ITT) Analysis

We conducted ITT analysis with 15 cancer survivors with missing baseline or post MAAT‐Class data using linear interpolation (See Table 1 for descriptions of individuals with missing data). This was done to evaluate if their inclusion in analyses would alter results. As seen in Table 2, PCI no longer attained a statistically significant improvement at post‐MAAT‐Class with interpolation. However, relaxation skills (*p* = 0.004), awareness of relaxation (*p* = 0.021) and coping confidence (*p* = 0.001) did significantly improve, with Perceived Cognitive Abilities (PCA) and Comments from Others (CFO) attaining marginal significance. Eight of the 15 individuals with missing (interpolated) data reported with a mean baseline PCI score of 43.20 (sd = 13.82) which is about 0.5 sd's above the mean baseline score of the sample with complete data (*N* = 22). All eight did not report any post‐MAAT class data, thus reducing baseline to post‐MAAT‐Class differences on PCI with a ceiling effect. Likewise, four individuals with interpolated data only provided post‐MAAT‐Class data on PCI with a low mean score of 23.63 (sd = 13.82) at the post‐MAAT‐Class time point which exceeds > 0.5 sd of baseline PCI scores in the sample with complete data (*N* = 22). Taken together, including data of 15 individuals may have altered PCI outcomes, but had negligible effects on others.

## 4 Discussion

Our observed clinical outcomes in this small sample of long‐term survivors participating in MAAT‐Class demonstrate improvements in cognitive symptoms (PCI), impact on QoL (IQoL), self‐regulation/relaxation skills and coping. MAAT‐Class has the potential to be a helpful educational intervention for CRCI that can efficiently reach survivors reporting CRCI, may help alleviate supportive care wait times and can potentially help individual survivors determine with their healthcare professional if more specialized cognitive care is necessary or desired. Age adjusted, statistically significant improvements in cognitive symptoms with a large effect (*d* = −0.79) were observed, with smaller effects on the QoL impact of cognitive symptoms (IQoL *d* = −0.39). Improvements were also observed in self‐regulation/relaxation skills, and confidence in coping with CRCI symptoms. There were no reported adverse events among participants. Overall, results suggest cancer survivors may derive benefit as a first step in CRCI management from this condensed educational program.

One strength of this clinical observational pilot study is that it reports results from a diagnostically diverse patient sample commonly seen for CRCI in supportive cancer care, although men were underrepresented (14%). By contrast, randomized controlled trials (RCT's) of CRCI treatments are most often conducted with diagnostically homogeneous groups of participants (e.g., breast cancer) who are also carefully screened for medical or psychiatric comorbidities that confound cognitive presentation (e.g., cancer recurrence, depression) [4]. RCT's may thus have limited generalizability to real‐world clinical settings where patients may present with multiple comorbidities that may influence cognitive function. While the cognitive and QoL outcomes presented here are limited to a small number of participants in an uncontrolled clinical observational study of MAAT‐Class, they suggest that an educational intervention could potentially yield positive outcomes in routine care.

### 4.1 Implications

The present results show positive effects among key CRCI outcomes for this small sample of consecutively referred patients. The MAAT‐Class educational format may allow a diverse set of patients with cognitive problems to engage survivorship care services more quickly and gain valuable knowledge of CRCI to help reduce symptom distress, enhance symptom coping, and engage with other survivors who may provide a source of support. It is important to note that MAAT‐Class is not proposed here to replace more comprehensive neuropsychological assessment, a full 8‐visit MAAT/CBT approach, or other cognitive rehabilitative or pharmacological efforts. Rather, it may act as a first step in more comprehensive CRCI supportive care. MAAT‐Class could also be adapted to hybrid in‐person and telehealth delivery which would likely improve survivor care access and satisfaction [12]. MAAT was designed to be telehealth delivered in an effort to reduce care disparities for survivors who live far from care centers, or who have transportation challenges in urban settings, or for who those who have exhausted savings or paid work leave with active cancer treatment [21]. Working with community outreach and engagement programs of comprehensive cancer centers to receive patient and family input in the design, build and deployment of research and health services can improve tailoring behavioral treatments in supportive care. e.g., MAAT‐Class could be modified to be co‐led by patient community leaders. Such efforts could help reduce access and utilization disparities in communities disadvantaged by race, socioeconomic status, or geographic location [22].

### 4.2 Limitations

There are substantive limitations to this observational clinical study. First, we cannot infer the efficacy of MAAT‐Class despite favorable outcomes and a somewhat representative sample of survivors with different cancers. There was no formal screening of participants, no comparison group, and there was a high amount of missing baseline and post‐MAAT‐Class data that may bias results toward those motivated to complete all outcome measures. The evaluable sample reported here is small, mostly female, and highly educated. This limits generalizability of results. Conducting an adequately powered RCT with a much larger, diagnostically and demographically diverse sample with usual care or other educational control arm would clarify efficacy. Such a trial could also help elucidate if individuals with different cancers and cancer therapies (e.g., CNS disease vs. hematologic malignancy) respond better or worse to MAAT‐Class. We did not conduct any medical record review. The final sample with complete data only had one person with primary brain tumor who reported they were about 1‐year post‐diagnosis. This individual unexpectedly scored 12 points higher (better) on the FACT‐Cog PCI at baseline than the mean of the sample. By contrast, one ovarian cancer survivor, 17 years post‐diagnosis, scored extremely low on PCI (almost 27 points lower than the group baseline). Survivors in this report were on average 4 years post‐diagnosis. We therefore lack information about MAAT‐Class effects on people in active treatment or immediately post‐treatment. Future research on MAAT‐Class offered to individuals completing active treatment or those in the acute post‐treatment phase of survivorship should be conducted to evaluate if it has beneficial effects on cognitive function or coping. A larger study sample could help determine which types of individuals respond better or worse to this educational intervention for those with reported cognitive problems.

Another limitation of this observational study was the lack of sample diversity. The evaluable sample with complete data had 19 women and only 3 men; the men scored higher on PCI at baseline (42.3) than women (32.72). The evaluable sample overall had post‐secondary and graduate education. Tailoring MAAT‐Class by obtaining survivorship and family caregiver input in its design (described above) could potentially broaden its applicability. In sum, a larger and properly powered RCT could evaluate efficacy and the influence of sex, education, race, time since diagnosis, treatment, and disease‐related factors on MAAT‐Class cognitive and coping outcomes.

Last, the primary outcome measure was a self‐report instrument (FACT‐Cog) and no objective neuropsychological assessment was used. A brief battery of neuropsychological testing outcomes could lend greater confidence to results [23]. However, the FACT‐Cog is widely used in clinical investigations and reflects patient experience of cognitive problems in daily life, whereas neuropsychological tests, administered under controlled conditions, may not [24]. Given MAAT‐Class is intended as a brief educational intervention as part of stepped care, a self‐report outcome measure of daily experience and QoL impact, such as the FACT‐Cog, is likely the most appropriate outcome measure [24].

## 5 Conclusion

MAAT‐Class has the potential to be broadly applied as a first‐step approach for addressing CRCI in cancer supportive care and survivorship services. The potential for simple implementation is high given the MAAT survivor workbook is published and readily available. However, our observations of cognitive and other outcomes in this report are limited to a small number of highly educated, mostly female longer‐term cancer survivors. More rigorous study of MAAT‐Class efficacy as an educational component of stepped survivorship care is needed in larger and more diverse communities. Continued development of educational support of CRCI to compliment ongoing behavioral and pharmacological CRCI treatment research [4] is encouraged for overall improved survivorship care.

## Funding

We gratefully acknowledge sources of support that include: NIH/NCI P30CA047904 (UPMC Hillman Cancer Center) and R01CA244673. Also, CA021765 (St Jude Children's Research Hospital Cancer Center, Dr. Charles Roberts, Principal Investigator) and the American Lebanese‐Syrian Associated Charities (ALSAC). The content is solely the responsibility of the authors and does not necessarily represent the official views of the National Institute of Health. This manuscript is the result of funding in whole or in part by the National Institutes of Health (NIH). It is subject to the NIH Public Access Policy. Through acceptance of this federal funding, NIH has been given a right to make this manuscript publicly available in PubMed Central upon the Official Date of Publication, as defined by NIH.

## Conflicts of Interest

Dr. Ferguson is the lead author of *Memory and Attention Adaptation Training (MAAT)* which is an evidence‐based cognitive‐behavioral therapy for treatment of cancer‐related cognitive impairment, published by Oxford University Press (OUP). Dr. Ferguson does receive modest annual royalties from OUP. Other authors report no current conflicts of interest.

## Data Availability

The data that support the findings of this study are available on request from the corresponding author. The data are not publicly available due to privacy or ethical restrictions.
